# Assessment of a Comprehensive Early Childhood Education Program and Cardiovascular Disease Risk in Midlife

**DOI:** 10.1001/jamanetworkopen.2021.20752

**Published:** 2021-08-20

**Authors:** Arthur J. Reynolds, Suh-Ruu Ou, Lauren Eales, Christina F. Mondi, Alison Giovanelli

**Affiliations:** 1 Human Capital Research Collaborative and Institute of Child Development, University of Minnesota, Minneapolis; 2 Institute of Child Development, University of Minnesota, Minneapolis; 3 Brazelton Touchpoints Center, Division of Developmental Medicine, Boston Children’s Hospital and Harvard Medical School, Boston, Massachusetts; 4 Division of Adolescent and Young Adult Medicine, Department of Pediatrics, University of California, San Francisco

## Abstract

**Importance:**

Educational attainment is an underrecognized factor associated with racial disparities in cardiovascular disease (CVD) risk. Early childhood programs providing comprehensive family services can promote educational and socioeconomic success, but few, if any, studies of large-scale programs have assessed their associations with midlife CVD risk and mediating factors.

**Objective:**

To examine the association between an early childhood program providing multisystemic services from ages 3 to 9 years with midlife CVD risk and whether years of education mediate these associations.

**Design, Setting, and Participants:**

Using a matched-group, quasi-experimental design, a cohort of Black and Hispanic children in the Chicago Longitudinal Study enrolled in an established early childhood program from 1983 to 1989 or the usual services were followed up for 30 years after the end of the intervention. A midlife survey on well-being was administered when the participants were aged 37 years. Analyses were conducted from September 1, 2020, to October 15, 2020.

**Intervention:**

The Child-Parent Center (CPC) Education Program provides school-based educational enrichment and comprehensive family services for 6 years, from ages 3 to 9 years (preschool to third grade [P-3]).

**Main Outcomes and Measures:**

General and hard Framingham Risk Scores (FRSs) were calculated from self-reported physical health and behavior profiles in the midlife Chicago Longitudinal Study survey. Years of education completed at age 34 years were measured primarily from administrative records.

**Results:**

There were 1539 participants in the original sample (1430 Black participants [92.9%]; 108 Hispanic participants [7.0%]; 1 White participant [0.1%]); 1104 of 1401 participants (78.8%) in the tracked sample completed a midlife survey on well-being by age 37 years, and 1060 participants had data available for analysis (mean [SD] age, 34.9 [1.4] years; 565 women [53.3%]), including 523 participants who grew up in high-poverty contexts. After adjusting for 17 baseline attributes and differential attrition via propensity score weighting, CPC preschool was associated with significantly lower general FRS (marginal coefficient, –2.2 percentage points [% hereafter]; 95% CI –0.7% to –3.6%; *P *= .004) and hard FRS (marginal coefficient, –1.6%; 95% CI –0.5% to –2.6%; *P *= .004), for a 20% reduction in cardiovascular disease risk. The program group was also less likely to have high-risk FRS status, including being in the top quartile of hard FRS (marginal coefficient, –7.2%; 95% CI, –0.3% to –11.6%; *P* = .02). Those who participated for 4 to 6 years (CPC P-3) had lower general FRS than those who participated for fewer years, but the difference was not significant (marginal coefficient, –1.2%; 95% CI, −2.5% to 0.2%; *P* = .09); longer participation was associated with placement in the median or higher risk category for general FRS (marginal coefficient, –7.9%; 95% CI –0.7% to –12.4%; *P *= .007) and hard FRS (marginal coefficient, –9.0%; 95% CI –0.6% to –11.4%; *P* = .02). Years of education completed by age 34 years accounted for up to 23% of the observed group differences for FRSs, including CPC preschool (general FRS, from –2.16% to –1.66%; difference = –0.5%) and P-3 (general FRS, from –1.16% to –0.71%; difference = –0.45%).

**Conclusions and Relevance:**

In this quasi-experimental design study, a comprehensive early childhood program for a community cohort at high risk of disparities was associated with lower CVD risk later in life. Corroborating previous research, years of education through college matriculation accounted for a sizable percentage of this association. Early childhood enrichment may contribute to CVD prevention.

## Key Points

**Question:**

Is a large-scale early childhood program providing comprehensive services from ages 3 to 9 years associated with midlife Framingham Risk Scores (FRSs), and is this association explained by educational attainment?

**Findings:**

In a matched-group cohort study that followed 1060 Black and Hispanic children from high-poverty communities to age 37 years, Child-Parent Center preschool participation was associated with a 20% reduction in cardiovascular disease risk, as measured by 30-year general FRS and hard FRS. The number of years of education by age 34 years partially mediated program-FRS associations, accounting for 23% of these observed and adjusted differences.

**Meaning:**

These findings suggest that a comprehensive and established multilevel early childhood program may promote cardiovascular health in midlife, which is associated with long-term risk of cardiovascular disease, the leading cause of death in the US.

## Teaser

This cohort study examines the associations between an early childhood program providing multisystemic services from ages 3 to 9 years and midlife cardiovascular disease risk and whether years of education attained mediate these associations.

## Introduction

As a leading socioeconomic determinant of health, educational attainment is underrecognized in the development and transmission of health disparities. In the US, Black individuals have rates of graduation at all levels of schooling that are 30% to 50% lower than those of White individuals.^1^ This is one explanation for why deaths from cardiovascular disease (CVD) and associated risk factors have racial disparities that are nearly identical to the education gaps.^2^ These disparities have been magnified in the coronavirus pandemic. In large part as a result of CVD risks such as hypertension, one-third of patients hospitalized or who have died from COVID-19 are Black, nearly 3 times their proportion in the population.^3^

Racial and ethnic disparities in health may be traceable to gaps in early learning associated with disproportionately lower access to high-quality preschool programs, schools, and health and community resource systems.^4^ Systemic discrimination and structural inequalities also are barriers to health and well-being.^2,3,4^ Designed to reduce disparities and increase educational attainment, early childhood programs provide enriched learning experiences, family supports, health services, and community outreach. Indeed, these programs have shown positive effects on many education outcomes that lead to greater economic well-being, crime prevention, and reduced health problem behaviors.^5,6^ Early prevention of CVD risks, however, has not been a major focus of study.

The few previous long-term studies^7,8,9^ on CVD risk have mixed results and are limited by their retrospective designs, small-sample trials with high attrition, and incomplete cardiovascular measurements. For example, the North Carolina early education Abecedarian Project evaluated a university-based efficacy trial in which only 72 participants, including 20 control male participants, were assessed in early midlife.^8^ The Ypsilanti, Michigan, HighScope Perry Preschool study at age 40 years also was a small efficacy trial and did not measure overall cardiovascular health.^9^ Although both cohort studies used random assignment, their generalizability and reproducibility are low.

In this study, we report the association between participation in an evidence-based, large-scale, multicomponent early childhood program in public schools and CVD risk at age 37 years. This is examined by analyzing Framingham Risk Scores (FRSs) for a predominantly Black cohort who participated in the Child-Parent Center (CPC) Education Program in Chicago, Illinois, from ages 3 to 9 years. Because school success is the primary goal of the program,^10^ the extent to which this association is explained by educational attainment also is tested.

## Methods

This cohort study was approved by the institutional review board of the University of Minnesota. Health examination data were approved by the institutional review boards of the Northwestern University Feinberg School of Medicine and the University of Minnesota. Informed consent was written and oral. This study generally follows the Strengthening the Reporting of Observational Studies in Epidemiology (STROBE) reporting guideline for cohort studies in organization, data procedures, and data interpretation.

The Chicago Longitudinal Study (CLS) is based on a quasi-experimental, matched-group, prospective, cohort design and includes 1539 participants born in 1979 and 1980 who attended early childhood programs in the Chicago Public Schools beginning in September 1983.^10,11,12^ Data from a variety of survey, interviews, and administrative records have been collected over the course of 4 decades. The CPC group included 989 participants enrolled at ages 3 or 4 years who continued participating until age 9 years. The same-age comparison group of 550 participants attended randomly selected schools receiving the usual early childhood program (full-day kindergarten), most without preschool. The demographic profiles of the groups were similar,^10^ with both groups growing up in high-poverty neighborhoods and similar numbers of Black and Hispanic participants (eTable 1 in the Supplement).

From ages 32 to 37 (August 20, 2012, to July 18, 2017; mean [SD] age, 34.9 [1.4] years), 1104 participants in the cohort (71.7% of the original sample of 1539 participants) completed a life course survey or interview of approximately 2 hours in length on experiences in education, employment, family life, and many dimensions of health. After accounting for deceased individuals and those who did not have sufficient information to be located, the effective response rate was 78.8%, or 1104 of 1401 participants in the tracked sample (Table 1). To corroborate and expand the survey or interview, an in-person health examination was conducted for a subsample of 301 participants (27.3% of the survey or interview sample) from ages 37 to 39 years (March 24, 2017, to December 21, 2019) at the Department of Preventive Medicine Clinic, Feinberg School of Medicine at Northwestern University, Chicago, Illinois.

**Table 1.** Characteristics of CPC Education Program and Comparison Groups in the Chicago Longitudinal Study: Original and Follow-up Sample at Age 37 Years

Study category	Total sample	Program group^a^	Comparison group^a^
Program participation at start of study, No. of participants^b^			
Original sample	1539	989	550
Preschool participation	1073	989	84
CPC preschool	989	989	0
2 y of preschool	534	534	0
Full-day kindergarten	1142	592	550
Part-day kindergarten	397	397	0
CPC school-age (1st to 3rd grade)	850	684	166
Duration of school-age program, mean (SD), y^c^	1.16 (1.17)	1.43 (1.09)	0.68 (1.16)
4-6 y of CPC (P-3)	553	553	0
Total time in CPC program, mean (SD), y^d^	2.78 (1.97)	3.95 (1.23)	0.68 (1.16)
Participants lost after program, No.			
Moved or not located^e^	363	215	148
Deceased before 2012	72	49	23
Follow-up interview at age 37 y			
Original sample who completed interview or survey, participants, No. (%)	1104 (71.7)	725 (73.3)	379 (68.9)
Framingham Risk Score calculated, participants, No.	1060	702	358
Tracked (available) sample who completed, No. of participants/total No. (%)	1104/1401 (78.8)	725/897 (80.8)	379/504 (75.2)
Select baseline attributes for follow-up sample, participants, No. (%)			
Black	991 (93.5)	653 (93.0)	238 (94.4)
Women	565 (53.3)	389 (55.4)	176 (49.2)
Men	495 (46.7)	313 (44.6)	182 (50.8)
Family risk index, mean (SD)^f^	4.44 (1.79)	4.43 (1.75)	4.47 (1.86)
Birth weight, lb, mean (SD)	6.80 (1.25)	6.83 (1.25)	6.74 (1.26)
Child welfare services	37 (3.5)	22 (3.1)	15 (4.2)
Mother attended some college or more	131 (12.4)	93 (13.2)	38 (10.6)
Reside in high poverty neighborhood^g^	528 (49.8)	395 (56.3)^h^	133 (37.2)
Family income <130% of federal poverty level	878 (82.8)	581 (82.7)	297 (83.0)

Abbreviation: CPC, Child-Parent Center.

SI conversion factor: To convert pounds to kilograms, multiply by 0.45.

^a^ Cases for program participation cover the 6-year period (1983-1989) that defines enrollment in the CPC program.

^b^ The comparison group participated in a full-day kindergarten program, and 84 participated in Head Start preschool; 109 parents in the comparison group reported their child participated in other childcare or education in preschool, and 176 cases in the comparison group received services in CPC kindergarten. They are not part of the original program group. Some cases in the comparison group participated in the school-age program because it was open to any child enrolled in elementary school. Fifteen children in the CPC intervention group were enrolled in the alternative full-day kindergarten.

^c^ The duration of the school-age program was 0 to 3 years.

^d^ The duration of the CPC program was 0 to 6 years.

^e^ These categories account for attrition from the original study sample of 1539. Cases were lost during postprogram years because they moved from Chicago, Illinois, and could not be located, were deceased, or either did not have sufficient identifying information to track, refused to participate or were incarcerated (other). The number of deceased participants by 2019 was 97. Baseline attributes were not weighted by inverse propensity score weighting attrition. Weighting reduced the displayed group differences.

^f^ The family risk index refers to parents’ education and income, with a score range of 0 to 8.

^g^ A high-poverty neighborhood is one where greater than or equal to 40% residents live in poverty.

^h^ *P* = .04.

### Framingham Risk Scores

On the basis of survey and interview responses (via telephone, in person, or online) to a variety of questions on physical health, we calculated for the participants with available data the 30-year hard FRS (H-FRS; ie, coronary death, myocardial infarction, or stroke) and general FRS (G-FRS), which also includes heart failure or disease, coronary insufficiency, transient heart attack, and angina.^13^ These estimated probabilities included the following factors: age at survey assessment, male sex, systolic blood pressure or treated hypertension, diabetes, current smoker, and body mass index (BMI; calculated as weight in kilograms divided by height in meters squared) in lieu of cholesterol levels (see the eAppendix in the Supplement).^13,14,15,16^ These reports correlated highly with FRS assessed by in-person examination of a CLS subsample (*r* = 0.85; eAppendix in the Supplement). Further CLS analyses of BMI and validation procedures are available elsewhere.^16^ FRS is the most widely used risk indicator for clinical estimation of CVD, and it performs similarly between White and Black adults.^13,17^ To signify high-risk groups, we defined 2 dichotomous thresholds at the median and top quartile of each score.

### CPC Program

CPC, which was developed in 1966 during the War on Poverty era, provides comprehensive educational and family support services from preschool to third grade (P-3) for children and families facing barriers to equal opportunity associated with poverty, discrimination, and racial segregation. The goal is to promote educational success leading to higher educational attainment and, ultimately, to greater economic well-being and health.^10,11,12,18^ A recent study^16^ of BMI in midlife showed benefits, especially for those most at risk of experiencing disparities. Besides educational and intensive enrichment for children, a broad array of family services, home visits, health and nutrition workshops and services, and community outreach were implemented. Key elements include small classes and activity-based learning, collaborative leadership, family engagement, and alignment of instruction across ages.^5,6,10,18^ After 1 or 2 years of part-day preschool at ages 3 and 4 years, continuing services are provided in the 20 CPC schools.

In CLS, the number of years of participation ranged from 0 (comparison group) to 6 (ages 3 to 9 years), with a mean (SD) among the CPC group of 3.95 (1.20) years. Our focus was on 2 comparisons: preschool and school-age (first to third grade) separately vs the no-CPC group, and 4 to 6 years of the total program (CPC P-3) vs lesser or no program participation (0 to 3 years). Complementing the latter, total years of intervention also was examined.

### Statistical Analysis

The model was estimated by linear and logit regression with inverse propensity score weighting^19,20^ to adjust for differential attrition (eAppendix in the Supplement).^6,10,16^ CPC preschool and school-age participation were assessed separately from the CPC P-3 group. The covariates were 17 multilevel baseline characteristics (eAppendix in the Supplement). Logit regression coefficients for dichotomous outcomes were transformed to marginal effects (coefficients) via the probit method. The outcomes on a continuous probability scale (30-year G-FRS and H-FRS) are reported as percentages (proportions), and differences are reported in percentage points (marginal coefficients). Mediation via educational attainment was tested by the difference-in-difference method.^21^ This estimates the percentage of observed differences in the main comparison that is associated with or explained by the focal mediator. Years of education completed by age 34 years and related educational attainment measures were obtained from administrative records and self-reports. Analyses were conducted in SPSS statistical software version 26 (IBM). *P* < .05 (2-tailed tests) denoted significance. This is conservative given prior research. Program interactions by sex, family risk indicators (eg, maternal low educational attainment or low household income; range, 0-8 indicators) by age 3 years, and neighborhood poverty (defined as ≥40% residents residing in impoverished households) were assessed. The latter 2 were measures from school records, family surveys, and the 1980 US Census (eAppendix in the Supplement). Data were analyzed from September 1, 2020, to October 15, 2020.

## Results

There were 1539 participants in the original sample (1430 Black participants [92.9%]; 108 Hispanic participants [7.0%]; 1 White participant [0.1%]). Of the 1104 survey or interview respondents, 601 (54.4%) were female, 1032 (93.5%) were Black, and the mean (SD) number of family risk factors during childhood was 4.4 (1.8) (Table 1). For the population of 1060 participants with FRS data (702 program participants and 358 comparison participants; 565 women [53.3%]), the mean (SD) G-FRS was 19.7% (11.1%), and the mean H-FRS was 11.3% (8.2%). Men were at higher risk than women on both indicators, and family demographic risk variables and neighborhood poverty (523 participants grew up in high-poverty contexts) were positively associated with FRSs (eTable 1 in the Supplement). The normal CVD risk for individuals aged 35 to 37 years is 11% for G-FRS and 6% for H-FRS.^13^

### CPC and CVD Risk

As shown in Table 2 and after baseline and inverse propensity score weighting attrition adjustment, CPC preschool participation was associated with significantly lower G-FRS (marginal coefficient, –2.2 percentage points [% hereafter]; 95% CI, –0.7% to –3.6%; 16% reduction; *P *= .004) and H-FRS (marginal coefficient, –1.6%; 95% CI, –0.5% to –2.6%; 15% reduction; *P *= .004). This pattern was also found for median or higher 30-year risk for CVD (G-FRS marginal coefficient, –11.4%; 95% CI, –3.3% to –16.1%; 19% reduction; *P* < .001), H-FRS (marginal coefficient, –10.0%; 95% CI, –1.5% to –13.4%; *P* = .003), and percentage in the top risk quartile of H-FRS (marginal coefficient, –7.2%; 95% CI, –0.3% to –11.6%; 26% reduction; *P* = .02). The overall mean risk reduction was 20%. The CPC group narrowed the gap in normal FRS risk probability by 12%. Among women, the CPC group narrowed the gap in normal risk probability by 18% (eAppendix in the Supplement).

**Table 2.** FRS by Age 37 Years for CPC Preschool, School-Age, and Preschool to Third Grade Groups, Adjusted for Baseline Attributes and IPW Attrition^a^

Outcome and sample group	FRS, %^b^												
	CPC preschool groups				CPC school-age groups				CPC P-3				Dosage partial correlation
	Intervention (n = 702)	Comparison (n = 358)	Difference	*P* value	Intervention (n = 606)	Comparison (n = 454)	Difference	*P* value	Intervention 4-6 y (n = 401)	Comparison <4 y (n = 659)	Difference	*P* value	
Total sample (n = 1060)													
General FRS	19.0	21.2	–2.2	.004^c^	19.5	19.9	0.4	.54	19.1	20.3	–1.2	.09^d^	–0.09^c^
Median or higher risk	48.1	59.5	–11.4	<.001	52.1	54.4	–2.3	.47	46.6	54.5	–7.9	.007^c^	–0.12^c^
Top 25% risk	23.5	27.4	–3.9	.17	26.9	24.9	2.0	.43	25.0	26.0	–1.0	.69	–0.06
Hard FRS	10.8	12.4	–1.6	.004^c^	11.8	11.4	0.4	.43	10.9	11.7	–0.8	.10^d^	–0.11^c^
Median or higher risk	50.9	60.9	–10.0	.003^c^	53.4	56.8	–3.4	.30	47.8	56.8	–9.0	.02^c^	–0.10^c^
Top 25% risk	21.0	28.2	–7.2	.02^c^	28.8	23.8	5.0	.048^c^	24.6	26.0	–1.4	.59	0.08^d^
Women (n = 565)													
Full FRS	14.4	17.4	–3.0	<.001^c^	16.2	16.4	–0.2	.82	14.6	16.3	–1.7	.02^c^	–0.13^c^
Median or higher risk	28.8	42.1	–13.3	.001^c^	35.5	36.6	–1.1	.78	29.6	35.6	–6.0	.11	–0.14^c^
Top 25% risk status	11.7	14.2	–2.5	.38	12.8	13.9	–1.1	.69	11.6	13.2	–1.6	.56	–0.06
Hard FRS	7.1	9.0	–1.9	.001^c^	8.2	8.3	–0.1	.81	7.1	8.3	–1.2	.02^c^	–0.13^c^
Median or higher risk	19.0	37.5	–18.5	.004^c^	30.2	32.4	–2.2	.56	26.3	32.1	–5.8	.12	–0.12^c^
Top 25% risk status	5.2	13.1	–7.9	.01^c^	11.9	10.5	1.4	.71	7.5	10.9	–3.4	.18	–0.10^c^
Men (n = 495)													
Full FRS	23.1	24.9	–1.8	.11	25.1	23.9	1.2	.29	23.7	24.5	–0.8	.44	–0.08^c^
Median or higher risk	70.6	76.4	–6.0	.09^d^	70.8	74.1	–3.3	.35	67.2	74.6	–7.4	.03^c^	–0.10^c^
Top 25% risk status	35.6	40.1	–4.4	.30	42.6	37.0	5.6	.15	39.5	39.5	0	>.99	–0.05
Hard FRS	14.0	15.6	–1.6	.08^d^	15.7	14.7	1.0	.23	14.7	15.3	–0.6	.44	–0.08^c^
Median or higher risk	81.4	83.5	–2.1	.50	76.0	83.8	–7.8	.08^d^	77.1	83.1	–6.0	.03^c^	–0.07^c^
Top 25% risk status	36.5	42.9	–6.4	.13	47.7	38.4	9.3	.02^c^	43.2	42.1	1.1	.78	0.07^d^

Abbreviations: CPC, Child-Parent Center; FRS, Framingham Risk Score; IPW, inverse propensity score weighting; P-3, preschool to third grade.

^a^ Values are adjusted for 17 baseline covariates (see main text and eAppendix in the Supplement) and IPW attrition. The top rows (general FRS and hard FRS) are probability values converted to percentages for 30-year risk. The difference is the adjusted marginal outcome. Preschool and school-age comparisons were estimated jointly. The CPC P-3 comparison was estimated separately and compares participation in the total program for 4 to 6 years vs lesser or no participation. Comparison group means and rates are unadjusted values. Adjusted sample size counts of CPC groups for selected outcomes were 338 participants for G-FRS (preschool) median or higher risk and 165 participants for top 25% risk. Respective sample sizes for the P-3 group were 187 and 100 participants. To be consistent with continuous outcomes (general or hard FRS), dichotomous outcomes for logit regression were converted to marginal coefficients in percentage points. Partial correlations are polyserial and biserial coefficients for 0 to 6 years (ages 3 to 9 years) adjusted for sex, race/ethnicity, family risk index, neighborhood poverty, attrition IPW, and lack of continuous scale properties.

^b^ Differences are shown in percentage points.

^c^ *P* < .05.

^d^ *P <* .10.

Although school-age services were not associated with most measures, participation for 4 to 6 years was consistently associated with lower FRSs, especially for participants with median or high risk for G-FRS (marginal coefficient, –7.9%; 95% CI, –0.7% to –12.4%; *P *= .007) and H-FRS (marginal coefficient, –9.0%; 95% CI, –0.6% to –11.4%; *P* = .02). Those who participated for 4 to 6 years (CPC P-3) had lower G-FRS than those who participated for fewer years, but the difference was not significant (marginal coefficient, –1.2%; 95% CI, −2.5% to 0.2%; *P* = .09). Group percentages in the top quartile risk were equivalent. Also shown in Table 2 is an inverse dose-response association between years of intervention (0 to 6) and FRS. The partial correlations are corrected for range restriction and document a pattern of association (*r* = –0.09 to –0.14) that is consistent with findings for the 4- to 6-year group. These results were unaffected by the addition of covariates, including kindergarten school readiness scores and CPC by risk factor interactions.

No differences in CPC associations were found for any subgroup. Given the differential risks for women and men, we report results for these groups in Table 2. The pattern of group differences favored women. We further note that alternative models with and without propensity score weighting consistently showed that the pattern of findings for all groups was robust (eTable 2 in the Supplement).

### Mediation by Educational Attainment

As the most comprehensive indicator, years of education completed by age 34 (mean [SD], 12.6 [2.1] years) was the strongest attainment mediator of the observed association between CPC participation and CVD risk. The Figure displays group differences for the broader G-FRS (patterns for H-FRS were identical) before and after adjustment for 3 attainment measures. Of the overall adjusted group difference, years of education accounted for 23% of this difference (preschool, from –2.16 to –1.66 percentage points [% hereafter] [difference, –0.5%]; CPC P-3, from –1.16% to –0.71% [difference, –0.45%]). This was similar for median risk. The other narrowly defined attainment measures accounted for 10% to 11% of program group differences (Figure). As a direct factor, each additional year of education was associated with a 1–percentage point decrease in G-FRS. Shorter-term kindergarten through grade 12 achievement test measures did not provide additional mediating power.

**Figure.**
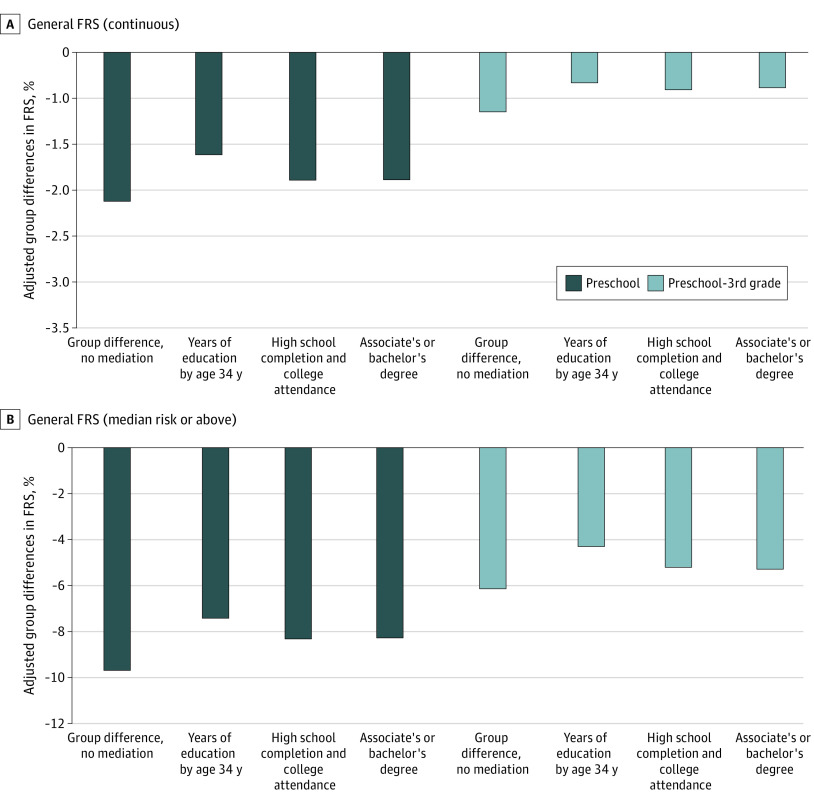
Group Differences in Framingham Risk Score (FRS) Before and After Educational Attainment Mediators Figure shows adjusted group differences in general FRS (G-FRS; main outcomes) for 2 Child-Parent Center (CPC) measures before and after accounting for educational attainment mediators. Assessed separately, the mediators were years of education by age 34 years, high school completion and college attendance (2 dichotomous variables), and earned associate’s or bachelor’s degree (1 dichotomous variable). For example, the inclusion of years of education reduced the preschool group difference in G-FRS from 2.16 percentage points to 1.66 percentage points (a reduction of 23.1%). All baseline covariates and inverse propensity score weighting attrition were included in the models. Results for hard FRSs were nearly identical (data not shown).

## Discussion

In a time of heightened health risks from the coronavirus pandemic, preventing CVD is an important goal,^3^ but it has been understudied in both cardiology and early intervention research. Extending on previous findings,^10,11,12^ our study documents for the first time, to our knowledge, an association between the CPC program and reduced CVD risk in midlife. The observed reductions over the comparison group in high-risk FRS status were sizable and averaged 20%. The largest reductions for the CPC preschool associations were the proportion in the top quartile of H-FRS (26% reduction) and the median risk or higher risk category of G-FRS (19% reduction). For the CPC P-3 group, reductions for the median risk or higher risk categories were 16% and 15%, respectively.

Because these findings are for an existing large-scale early childhood program,^5,16,18^ they are more generalizable to contemporary services than the mixed evidence from prior efficacy trials and retrospective studies.^7,8,9^ The consistency of findings across subgroups suggests that the intervention provides a generalizable preventative response for midlife CVD risk. Although this should be explored in other contexts and programs, the comprehensive services, intensive enrichment, and longer duration appear to be essential. These align well with recommendations to promote health equity and disease prevention.^22^ To our knowledge, no prior early childhood studies have assessed dosage response (Table 2). Robustness of findings across different model specifications and covariates supports this interpretation.

The absence of inverse associations of the CPC school-age program with FRS is consistent with prior studies of related outcomes in the CLS project.^6,10,11,12,16,23^ Although the school-age component is less intensive and comprehensive than the preschool component, the findings reveal that the timing and duration of services together make more of a difference. This is shown by the pattern of findings for the P-3 group (Table 2). This pattern has also been documented in prior studies.^6,10,11,12^ Further analysis by subgroups and testing of mediators will enhance understanding of these associations.

Although nearly all of the study participants are Black and grew up in low-income, urban neighborhoods, these findings provide evidence that early learning programs can contribute to the reduction of racial disparities in cardiovascular health.^2^ On the basis of the available norms of the FRS, which are more heavily weighted to White, middle-class samples, the CPC group narrowed the gap in normal FRS risk probability by 12%. Among women, the CPC group narrowed the gap in normal risk probability by 18% (eAppendix in the Supplement). Given the 30-year time span, this reduction is notable but is also likely to be conservative. Further matching by neighborhood and family poverty would identify the unique contribution to reductions in racial disparities.

Educational attainment accounted for a substantial share of the observed associations with Framingham risk. This is consistent with prior studies of other life course outcomes, CVD mortality, and epidemiology^5,6,10,24^ and with the primary goal of the CPC program. However, education was only partially effective as a mediator given that only approximately one-quarter of the group differences were explained, and program participation usually remained significant even after education was added. As a confirmatory study, we did not test other mediators that may contribute to program CVD risk reduction. Because education is a component of socioeconomic status, income, employment, and occupational prestige are measures that warrant further analysis. Identified factors associated with educational attainment, including school achievement and performance, socioemotional adjustment, family involvement, school and community support, and justice system involvement, may also have important roles.^5,6,10,22^ Determinants and indicators of community, social, and economic support that affect health and well-being include racial discrimination, structural inequality, poverty, and segregation.^25,26,27,28^

Our focus was on long-term educational success, the primary goal of the program. Investigation of other mediators was beyond the scope of the present study. Among the most salient extensions that show supportive evidence for health behaviors and economic well-being include socioemotional learning and self-control, motivation, cognitive skills, school and community support, and health literacy.^6,29,30^ Separate and joint estimation of the contribution of these measures are warranted.

### Limitations

Three limitations of this study are that the results may not generalize to programs providing services of lesser quality and comprehensiveness. First, the sample characteristics also were homogeneous and may not generalize beyond low-income Black populations. Second, our measures of FRS used BMI as a proxy for lipid levels, which may limit precision. However, this approach is a common practice,^11^ and our self-reports were validated by direct examination. In support of robustness of findings, in-person examination using BMI as a stand-in for cholesterol and measured cholesterol from blood samples were highly correlated (*r* = 0.85). Third, alternative measures of CVD risk warrant testing in our predominantly urban Black cohort.^14,15,17^ One such measure is the American Heart Association’s Ideal Cardiovascular Health (Life’s Simple 7).^31,32,33^ With these measures, complementary and alternative approaches to mediation may include complex paths of direct and indirect influences.

## Conclusions

In conclusion, a school-based early childhood program providing multisystemic services showed evidence of 30-year CVD risk reduction compared with the usual educational experiences in a large urban school district. That continuing services in early elementary school demonstrated enhanced risk reduction suggests that longer duration programs can support the long-term health of participants who face persistent disparities across health service and education systems.

## Notes

Supplement. **eAppendix. **Supplemental Methods
eReferences.
**eTable 1.** Chicago Longitudinal Study Sample Characteristics by Attrition Status **eTable 2.** Alternative Models of Child-Parent Center (CPC) and Comparison Group Differences for 30-Year General and Hard Framingham Risk Scores (FRS) by Age 37
