# Supplementary material for: Assessment of a Comprehensive Early Childhood Education Program and Cardiovascular Disease Risk in Midlife
Source: JAMA Netw Open. 2021 Aug 20;4(8):e2120752. doi: 10.1001/jamanetworkopen.2021.20752 (PMC8379657; doi:10.1001/jamanetworkopen.2021.20752)
Supplement: Supplement. — eAppendix. Supplemental Methods eReferences. eTable 1. Chicago Longitudinal Study Sample Characteristics by Attrition Status eTable 2. Alternative Models of Child-Parent Center (CPC) and Comparison Group Differences for 30-Year General and Hard Framingham Risk Scores (FRS) by Age 37 [file jamanetwopen-e2120752-s001.pdf]

Supplemental Online Content

Reynolds AJ, Ou SR, Eales L, Mondi CF, Giovanelli A. Assessment of a comprehensive early childhood education program and cardiovascular disease risk in midlife. *JAMA Netw Open*. 2021;4(8):e2120752. doi:10.1001/jamanetworkopen.2021.20752

eAppendix. Supplemental Methods

eReferences.

eTable 1. Chicago Longitudinal Study Sample Characteristics by Attrition Status

eTable 2. Alternative Models of Child-Parent Center (CPC) and Comparison Group Differences for 30-Year General and Hard Framingham Risk Scores (FRS) by Age 37

This supplemental material has been provided by the authors to give readers additional information about their work.

© 2021 Reynolds AJ et al. *JAMA Network Open.*

eAppendix. Supplemental Methods

Program Description

CPC provides comprehensive, multi-systemic services in education and schooling, family support, health, and community outreach.[1,2] Under the direction of the Head Teacher at each site and in collaboration with the Principal, CPC is designed to enhance school readiness and achievement, promote parent involvement and engagement in the school and community, and enhance socio-emotional learning with an emphasis on self-control, self-efficacy, and personal responsibility. Breakfasts and lunches are provided, school nurses work with families on site, and referrals to health centers, speech therapy and other supports are provided. CPCs are in a stand-alone school or center in which all children receive services.

Based on the goals and foci of the program, participants' experiences, and impacts to date,[3-9] the program is expected to promote cardiovascular health and reduced cardiovascular disease (CVD) risk through four pathways:

a. educational success and attainment
b. self-control and self-efficacy behaviors
c. health literacy and practices
d. social support and engagement.

These paths are interconnected yet, based on the theory of the program, educational success and attainment is fundamental.[1,3,4] Operationally, after a part-day program (3 hours, 5 days per week) at ages 3 and/or 4 in small classes with child-teacher ratios of 17:2, CPC K–3rd components provide reduced class sizes (maximum of 25), teacher aides for each class, health services, continued parent involvement opportunities, and enriched classroom environments for strengthening language and literacy, math, science, and social-emotional learning. To promote wholistic well-being, including physical health, each center has a parent resource room and family program run by the Parent-Resource Teacher in collaboration with the School-Community Representative.

The later conducts home visits, engages parents in the school, mobilizes resources in the community and provides referrals to health, employment and job training, and related services. Parent workshops and trainings are a predominant element of the program, and they most frequently include child development, health literacy, nutrition, financial literacy, and personal development topics. GED courses are often provided on site and parents volunteer in the classroom and in community organizations. Given the physically located resource room in the centers, peer support among parents and family members is another key feature. Many reports are available describing program history, key elements and principles, and implementation.[1-5,7,10]

Calculation of Framingham Risk Scores (FRS)

The CLS midlife survey from ages 32 to 37 (August 20, 2012 to July 18, 2017; mean age: 34.9 years) assessed many dimensions of physical health, metabolic status, and health behaviors. We measured FRS, the most widely used CVD indicator for clinical prediction.[11-15] We focused on 30-year risk scores, the prediction over the next 30 years that individuals 20 to 59 years of age and who are free of CVD and cancer will have a major CVD event or die from the disease. To calculate 30-year "general" (G-FRS) and "hard" (H-FRS) metrics, we used the Framingham Heart Study's scoring calculator (https://framinghamheartstudy.org/fhs-risk-functions/cardiovascular-disease-30-year-risk/) as documented in the published analysis and findings.[12] The prediction factors are as follows and were measured for 1060 study participants:

1. Male sex (a risk factor)
2. Age at the time of interview in years and months.
3. Systolic blood pressure (report of hypertension/treatment used if value not provided)
4. Treated for hypertension
5. Diabetes (reported in interview as being diagnosed)
6. Smoker (with any frequency currently)
7. Body Mass Index (calculated from height and weight) in lieu of cholesterol levels.

The score is a probability value between 0 and 1that an individual with his/her 7-factor profile will have CVD or a serious or fatal cardiovascular event. For the BMI-based scores, the CLS sample means were

© 2021 Reynolds AJ et al. *JAMA Network Open.*

19.7% (G-FRS) and 11.3% (H-FRS). The normal and optimal values, respectively, for the ages of study members are 11.0% and 8.9% (G-FRS) and 5.6% and 4.3% (H-FRS). For women and men, the probability values for the CLS sample in comparison to FRS norms were as follows:

	Women	Men	Total
**A. G-FRS Probability**			
CLS estimated risk	15.7	24.2	20.0
Normal risk	8.4	14.1	11.3
Optimal risk	6.7	11.3	9.0
**B. H-FRS Probability**			
CLS estimated risk	7.9	15.0	11.5
Normal risk	3.7	7.8	5.8
Optimal risk	2.8	6.1	4.5

A large literature describes the background, development, findings, and use of the FRS in many populations.[13-23] For example, scores predict equally for White and Black adults.[13] The calculated scores correlated highly with in-person FRS exam scores (r = .85; N = 286) regardless of whether cholesterol or BMI was included. The in-person exam had a total sample of 301 participants and was completed at the Northwestern University Department of Preventive Medicine (Feinberg School of Medicine) in Chicago from March 24, 2017 to December 21, 2019. We further note that self-reported BMI was highly corrected with in-person exam measurement (r = .85) for the 286 participants with both sets of scores. Body composition and waist circumference were also highly correlated with BMI reports.

Educational Attainment Mediators at Age 34

Our primary measure for assessing mediation was years of completed education (range = 7 to 22) by May 2014 (mean age 34.1 years). The value 12 denotes a high school diploma or equivalent credential; and 14, 16, 18, and 20 Associate's through Doctorate degree (Post-Doctorate is possible). The two alternative indicators were (a) high school completion by diploma or equivalent (GED) and any college attendance (versus high school dropout), and (b) earned Associate's or Bachelor's degree or higher).

Education is one of the most comprehensively measured indicators in the CLS.[4,6,8,10] Data were obtained from many sources, including the Chicago Public Schools (CPS) yearly up to 2003, Illinois Department of Child and Family Services (DCFS) in 2003 and 2008, Illinois Shared Enrollment and Graduation Consortium (ISEG) yearly from 2002 to 2009, City Colleges of Chicago in 2001-2002, Illinois GED Testing Program in 2006, and the National Student Clearinghouse (NSC) in May 2014. These administrative records were supplemented by self-reports at ages 20, 22-24 and 32-34 (midlife interview), and in tracking for the midlife interview. The cut-off date for measurement was May 31, 2014.[4]

For high school completion status, 114 participants reported postsecondary education attendance, but they are missing on whether they completed high school via diploma or GED. Their types (high school graduation or GED) of high school completion were estimated based on available information from other data sources, including ISEG, NSC, CPS, and DCFS. For years of education, 2 participants are missing last grade they completed before they dropped out of school.

For the present study 1,025 of the 1,060 study sample members had a valid value for educational attainment at age 34. In the total CLS cohort, 1,473 participants had available data on any level of educational attainment. Of these, 1,397 were defined as being in the educational attainment sample at age 34. They met the following criteria:

a) Included in age 29 educational sample (n=1,382)
b) Had any information in the National Student Clearinghouse in May 2014 (n=634)
c) Completed age 35 survey by 5/1/2014 (n=321)
d) Not deceased by 2002 January.

Mediation Procedure

Following existing methodological practice[24,25] and prior CLS research,[1,6,8] we used the difference-in-difference model of mediation--referred to as the "percentage reduction" (PR) approach--to assess whether educational attainment accounted for at least part of the observed group differences. In this method, the

© 2021 Reynolds AJ et al. *JAMA Network Open.*

adjusted group difference with the hypothesized mediator ($B_{\_m}$) is subtracted from the adjusted group difference without the mediator B ("main effect model") and then expressed as a percentage reduction over the main effect model. Ranging from 0 to 100 percent, values larger than 100 percent are taken as 100 percent. Negative calculated values, often do to suppressor effects, are reported as 0. Thus the calculation is as follow:

$$PR = \frac{B - B_{\_m}}{B} \times 100$$

For example, if the "main effect" for G-FRS is 3 points and then is reduced to 2 points under the mediation model (e.g., educational attainment added), PR would be 33%. This would denote a sizeable reduction in the observed difference or partial mediation but far short of complete or full mediation. Of course, any one mediator would not be expected to make a large contribution given that multiple mediators are most likely and influences are both indirect and direct. The descriptive PR method is often a first step to more complex (but with many assumptions) methods ranging from path analysis to structural equation modeling via full information maximum likelihood estimation.[1,8]

Baseline Covariates for Estimating Impacts

Based on administrative records from multiple sources and parent surveys,[6,8,10] 17 variables were included as model covariates. They were measured primarily from birth to age 3 as baseline characteristics. Two significant differences were detected between groups: CPC participants grew up in higher poverty neighborhoods and their parents had higher rates of high school completion (but not college attendance). Sample attrition from the original cohort is shown in eTable 1. The covariates were as follows (all dichotomous with one exception):

1. Black race (vs. Hispanic and one non Hispanic White)
2. Female
3. Reside in single parent household
4. Parent dropped out of high school
5. Parent was under 19 years of age at child's birth
6. Family income 130% of the federal poverty level or below
7. Four or more children in the household
8. Parent not employed
9. Family received public aid (Aid to Families with Dependent Children [now TANF])
10. Reside in school attendance area with 60% or more of residents low income (<185% poverty level)
11. Family risk indicator was imputed (Variables 3-10)
12. Received child welfare services (0-3 years of age)
13. Adversity in the home environment retrospectively reported by participant (0-5 years of age)
14. Parent(s) attended college or postsecondary education (any length of time)
15. Reside in census track neighborhood of concentrated poverty (40% of residents at/below poverty)
16. Reside in census track neighborhood in which 10% or more of 25-year-olds and above have a BA.
17. Birth weight in pounds (1979-1980 birth records from Illinois Department of Health).

CPC preschool and school-age were included together as the program variables and CPC-P3 (4 or more years versus fewer) was included separately.

Inverse Propensity Score Weighting

Following previous CLS reports,[4,6,7,9] Inverse Propensity Score Weighting (IPW) was used to adjust for potential attrition bias. About one quarter of the cohort were missing on FRS either because they did not complete the survey or had insufficient information to calculate a score. IPW methods can reduced attrition bias arising from measurable factor influencing sample recovery status.[26] The regression models included the following weight variable:

$$W_i = 1/P_i$$

© 2021 Reynolds AJ et al. *JAMA Network Open.*

$$P_{i\,(SR)} = \text{Constant} + \mathbf{B}_j BD + \mathbf{B}_j HE + \mathbf{B}_j PR + \mathbf{B}_j SN + e$$

The predicted probabilities of sample recovery (SR; age 37 survey) were estimated by logit regression (linear regression yielded similar estimates) with 31 input predictors hypothesized or known to be important. These included birth outcomes and demographics (BD), home environment (HE), program (PR), school, and neighborhood factors (SN) such as poverty and the share of those aged . In the outcome regressions, this weight was applied such that individuals with higher weights were counted more heavily in program estimates, as they have lower probabilities of responding to the adult survey. The lower weighted were counted less. Standard errors are adjusted to account for these weights. Robustness analyses with this and alternative model are shown in eTable 2.

eReferences

1. Reynolds AJ. *Success in early intervention: The Chicago Child-Parent Center*. Lincoln: University of Nebraska Press, 2000.

2. Sullivan, L. M. *Let us not underestimate the children*. Glenview, IL: Scott Foresman, 1971.

3. Reynolds AJ, Hayakawa M, Candee, AJ, Englund, MM. *CPC P-3 program manual: Child-Parent Center Preschool-3rd Grade Program.* Minneapolis, MN: Human Capital Research Collaborative, University of Minnesota, 2016.

4. Reynolds AJ, Ou S-R, Temple JA. A multicomponent, preschool to third grade preventive intervention and educational attainment at 35 years of age. *JAMA Pediatrics.* 2018; 172(3):247-256.

5. Reynolds AJ, Temple JA, Robertson DL, Mann EA. Long-term effects of an early childhood intervention on educational achievement and juvenile arrest: A 15-year follow-up of low-income children in public schools. *JAMA.* 2001;285(18):2339-2346.

6. Reynolds, AJ, Temple, JA, Ou, S-R, Arteaga, IA, White BA. School-based early childhood education and age-28 well-being: Effects by timing, dosage, and subgroups. *Science. 2011; 333*(6040), 360-364.

7. Reynolds AJ, Temple JA, White, BA, Ou S, Robertson DL. (2011). Age-26 cost-benefit analysis of the Child-Parent Center early education program. *Child Development*, *82*(1), 379-404.

8. Reynolds AJ, Ou S-R. Paths of Effects From Preschool to Adult Well-Being: A Confirmatory Analysis of the Child-Parent Center Program. *Child Development.* 2011;82(2):555-582.

9. Reynolds AJ, Temple JA, Ou S-R, et al. Effects of a school-based, early childhood intervention on adult health and well-being. *Archives of Pediatrics & Adolescent Medicine.* 2007;161(8):730-739.

10. Chicago Longitudinal Study. *CLS user's guide: A study of children in the Chicago Public Schools*. Minneapolis: University of Minnesota, Institute of Child Development, 2005.

11. Armstrong AC, Jacobs DR, Gidding SS, et al. Framingham score and LV mass predict events in young adults: CARDIA study. *International Journal of Cardiology.* 2014;172(2):350-355.

12. Pencina MJ, D'Agostino RB Sr, Larson MG, Massaro JM, Vasan RS. Predicting the 30-year risk of cardiovascular disease: the Framingham Heart Study. *Circulation*. 2009;119(24):3078-3084. doi:10.1161/CIRCULATIONAHA.108.816694. See https://framinghamheartstudy.org/fhs-risk-functions/cardiovascular-disease-30-year-risk/

© 2021 Reynolds AJ et al. *JAMA Network Open.*

13. D'Agostino RB, Grundy S, Sullivan LM, Wilson P, for the CHD Risk Prediction Group. Validation of the Framingham Coronary Heart Disease Prediction Scores: Results of a Multiple Ethnic Groups Investigation. *JAMA.* 2001;286(2):180–187. doi:10.1001/jama.286.2.180

14. Khalili D, Hadaegh F, Soori H, Steyerberg EW, Bozorgmanesh M, Azizi F. Clinical Usefulness of the Framingham Cardiovascular Risk Profile Beyond Its Statistical Performance: The Tehran Lipid and Glucose Study. *American Journal of Epidemiology.* 2012;176(3):177-186.

15. Lloyd-Jones DM, Hong Y, Labarthe D, et al. Defining and Setting National Goals for Cardiovascular Health Promotion and Disease Reduction. *Circulation.* 2010;121(4):586-613.

16. Lloyd-Jones DM, Larson MG, Beiser A, Levy D. Lifetime risk of developing coronary heart disease. *The Lancet.* 1999;353(9147):89-92.

17. Lloyd-Jones DM, Larson MG, Leip EP, et al. Lifetime Risk for Developing Congestive Heart Failure. *Circulation.* 2002;106(24):3068-3072.

18. Lloyd-Jones DM, Wang TJ, Leip EP, et al. Lifetime Risk for Development of Atrial Fibrillation. *Circulation.* 2004;110(9):1042-1046.

19. Lloyd-Jones DM, Wilson PWF, Larson MG, et al. Lifetime Risk of Coronary Heart Disease by Cholesterol Levels at Selected Ages. *Archives of Internal Medicine.* 2003;163(16):1966-1972.

20. Lloyd-Jones DM. Cardiovascular Risk Prediction. *Circulation.* 2010;121(15):1768-1777.

21. Steyerberg EW, Vickers AJ, Cook NR, et al. Assessing the performance of prediction models: a framework for traditional and novel measures. *Epidemiology.* 2010;21(1):128-138.

22. Bosomworth NJ. Practical use of the Framingham risk score in primary prevention: Canadian perspective. *Can Fam Physician*. 2011;57(4):417-423.

23. D'Agostino RB, Sr., Pencina MJ. Invited Commentary: Clinical Usefulness of the Framingham Cardiovascular Risk Profile Beyond Its Statistical Performance. *American Journal of Epidemiology.* 2012;176(3):187-189.

24. MacKinnon DP. *Introduction to statistical mediation analysis*. New York: Erlbaum; 2008.

25. MacKinnon DP, Fairchild AJ, Fritz MS. Mediation analysis. *Annu Rev Psychol*. 2007;58:593-614. doi:10.1146/annurev.psych.58.110405.085542

26. Imbens GW, Wooldridge JM. Recent developments in the econometrics of program evaluation. *Journal of Economic Literature*. 2009*; 47*(1), 5-86.

© 2021 Reynolds AJ et al. *JAMA Network Open.*

eTable 1. Chicago Longitudinal Study Sample Characteristics by Attrition Status

Characteristics	N	Original sample (n=1539)	Study sample (n=1060)	Attrition sample (n=479)	Mean difference (Study-attrition sample) (P value)
Percent Female	1531	50.2	53.3	43.1	10.2* (<.01)
Percent Black	1539	93	93.5	91.9	1.6 (.28)
Child's birth weight in pounds	1539	6.8	6.8	6.8	0.0 (.85)
Family risk index (0-7) by child's age 3 [1]	1539	4.5	4.4	4.7	-0.3* (< .01)
Percent four or more risk factors by child's age 3 [1]	1539	72.9	71.4	76.2	-4.8 (.06)
Percent mother not completed high school by child's age 3 [1]	1539	54.3	52.4	58.5	-6.1* (.03)
Percent mother with some college experience by child's age 3 [1]	1539	12.2	12.4	11.9	0.5 (.87)
Percent single parent by child's age 3 [1]	1539	76.5	75.6	78.5	-2.9 (.22)
Percent mother not employed by child's age 3 [1]	1539	66.3	65.4	68.5	-3.1 (.24)
Percent ever reported receiving free lunch by child's age 3 [1]	1539	83.8	82.8	85.8	-3.0 (.16)
Percent ever reported receiving AFDC by child's age 3 [1]	1539	62.2	60.9	66.8	-5.9* (.03)
Percent with 4 or more children at home by child's age 3 [1]	1539	16.6	16.6	16.7	-0.1 (1.0)
Percentage children in school area in which 60% or more of children reside in low-income families	1539	76.0	75.4	77.2	-1.8 (.44)
Percent any child welfare case history by child's age 3 [1]	1539	4.1	3.5	4.4	-0.9 (.39)
Percent mother was teen at child's birth [1]	1539	16.2	15.4	18.0	-2.6 (.21)
Percent of all persons in census track at birth below the federal poverty level (1980 U. S. Census)	1538	50.2	50.1	49.6	0.5 (.96)
Percent CPC preschool participation	1539	64.3	66.2	59.9	6.3* (.02)
Percent CPC school-age participation	1539	55.2	57.2	50.9	6.3* (.02)
Percent CPC extended participation	1539	35.9	37.8	31.7	6.1* (.02)

© 2021 Reynolds AJ et al. *JAMA Network Open.*

Note. Representative characteristics are reported with no weighting. 1. Means are reported after imputation for missing data using the Expectation-Maximization method. P values for percentages are based on Fisher's Exact Test. Sources of data include school records, parent surveys, state and local administrative data, and the U. S. Census (1980). CPC = Child-Parent Centers. AFDC = Aid to Families with Dependent Children (now Temporary Assistance for Needy Families). *95% CI does not contain zero.

© 2021 Reynolds AJ et al. *JAMA Network Open.*

eTable 2. Alternative Models of Child-Parent Center (CPC) and Comparison Group Differences for 30-Year General and Hard Framingham Risk Scores (FRS) by Age 37

	CPC Preschool Groups		CPC School-age Groups		P-3 (0-6y)	
Outcome/Sample group	Group diff	P value	Group diff	P value	Group diff	P value
**General FRS**						
1. Continuous score (Percentage)						
Unadjusted, No IPW	-2.5	.001	0.5	.530	-1.5	.031
Adjusted, No IPW	-2.0	.006	0.3	.666	-1.1	.089
Adjusted-IPW, Fixed effects	-2.5	.018	-0.2	.846	-1.1	.159
Adjusted by Double IPW	-2.1	.003	0.5	.515	-1.1	.071
2. Median or higher risk (≥17%)						
Unadjusted, No IPW	-11.9	.001	-1.0	.767	-8.3	.008
Adjusted, No IPW	-9.0	.007	-2.0	.511	-6.2	.034
Adjusted-IPW, Fixed effects	-11.5	.012	-5.2	.157	-5.6	.103
Adjusted by Double IPW	-10.5	.001	-0.8	.789	-6.3	.025
**Hard FRS**						
1. Continuous score (Percentage)						
Unadjusted, No IPW	-1.8	.001	0.4	.426	-1.1	.031
Adjusted, No IPW	-1.5	.006	0.3	.517	-0.8	.101
Adjusted-IPW, Fixed effects	-1.8	.018	0.1	.934	-0.8	.153
Adjusted by Double IPW	-1.5	.004	0.4	.429	-0.8	.074
2. Median or higher risk (≥9%)						
Unadjusted, No IPW	-10.3	.003	-1.9	.573	-8.4	.008
Adjusted, No IPW	-7.1	.022	-2.7	.345	-6.0	.029
Adjusted-IPW, Fixed effects	-12.0	.005	-5.8	.091	-6.3	.049
Adjusted by Double IPW	-8.5	.003	-1.3	.640	-5.8	.027

Note. Adjusted differences include 17 baseline covariates and, where noted, Inverse Propensity Score Weighting (IPW) attrition.

© 2021 Reynolds AJ et al. *JAMA Network Open.*

Preschool and school-age contrasts were estimated jointly. The CPC P-3 contrast was estimated separately and compares participation in the total program for 4-6 years versus lesser or no participation. To be consistent with continuous outcomes (General or Hard FRS), dichotomous outcomes for logit regression were converted to marginal coefficients in percentage points.
IPW is Inverse Propensity Score Weighting for attrition. Double IPW is the attrition weight * program selection weight (based on propensity score for participation) in either the preschool component or P-3 component (17 predictors for preschool and school-age; 18 for P-3).
Covariates were participant gender, race, and family risk index.

© 2021 Reynolds AJ et al. *JAMA Network Open.*
